# ABCC2 is associated with *Bacillus thuringiensis* Cry1Ac toxin oligomerization and membrane insertion in diamondback moth

**DOI:** 10.1038/s41598-017-02545-y

**Published:** 2017-05-24

**Authors:** Josue Ocelotl, Jorge Sánchez, Isabel Gómez, Bruce E. Tabashnik, Alejandra Bravo, Mario Soberón

**Affiliations:** 1 Instituto de Biotecnología, Universidad Nacional Autónoma de México. Apdo. Postal 510-3, Cuernavaca, Mor. 62210 Mexico; 2 Department of Entomology, University of Arizona, Tucson, Arizona USA

## Abstract

Cry1A insecticidal toxins bind sequentially to different larval gut proteins facilitating oligomerization, membrane insertion and pore formation. Cry1Ac interaction with cadherin triggers oligomerization. However, a mutation in an ABC transporter gene (ABCC2) is linked to Cry1Ac resistance in *Plutella xylostella*. Cry1AcMod, engineered to lack helix α-1, was able to form oligomers without cadherinbinding and effectively countered Cry1Ac resistance linked to ABCC2. Here we analyzed Cry1Ac and Cry1AcMod binding and oligomerization by western blots using brush border membrane vesicles (BBMV) from a strain of *P. xylostella* susceptible to Cry1Ac (Geneva 88) and a strain with resistance to Cry1Ac (NO-QAGE) linked to an ABCC2 mutation. Resistance correlated with lack of specific binding and reduced oligomerization of Cry1Ac in BBMV from NO-QAGE. In contrast, Cry1AcMod bound specifically and still formed oligomers in BBMV from both strains. We compared association of pre-formed Cry1Ac oligomer, obtained by incubating Cry1Ac toxin with a *Manduca sexta* cadherin fragment, with BBMV from both strains. Our results show that pre-formed oligomers associate more efficiently with BBMV from Geneva 88 than with BBMV from NO-QAGE, indicating that the ABCC2 mutation also affects the association of Cry1Ac oligomer with the membrane. These data indicate, for the first time, that ABCC2 facilitates Cry1Ac oligomerization and oligomer membrane insertion in *P. xylostella*.

## Introduction

Biological insecticides based on *Bacillus thuringiensis* (Bt) are important for insect control in agriculture. Bt bacteria produce different insecticidal proteins, like the three-domain-Cry toxins (3d-Cry) that have been used in insecticidal spray products for more than 50 years^1, 2^. Also, different *cry* genes have been introduced into the genome of important crops providing an effective way to control the damage of insect pests^2^. Cry toxins are highly specific, have proven to be innocuous to vertebrates and plants, and are biodegradable. However, evolution of insect resistance to Cry toxins threatens the efficacy of *Bt* toxins to control crop pests. In fact, resistance to many Cry toxins has evolved under laboratory and field conditions^3–9^. The most common mechanism for Cry toxin resistance is reduced binding of the toxin to insect gut membranes^10, 11^.

The 3d-Cry toxins constitute intestinal poisons that form pores in the midgut cell of different larvae, destroying these cells and killing the larvae^12^. Once susceptible larvae ingest the 3d-Cry protoxin, this protein is solubilized and activated by gut proteases. The protease resistant fragment or activated toxin, composed of the three-domain structure, undergoes a complex sequential binding events with different insect proteins leading to oligomerization, membrane insertion and pore formation, resulting in colloidal osmotic lysis of the midgut cells^13–15^.

In lepidopteran larvae the first binding event of activated Cry1A toxins is proposed to be a low affinity interaction with the highly abundant GPI-anchored-receptors, alkaline phosphatase (ALP) or aminopeptidase-N (APN)^15–18^. This interaction is proposed to concentrate the toxin in proximity to the brush border microvilli membrane of the midgut cells where the toxin binds, in a high affinity interaction, to the cadherin receptor^15^. The binding to cadherin promotes the proteolytic cleavage of the N-terminal end including helix α − 1 and part of helix α − 2, triggering the formation of an oligomeric structure^15, 19, 20^. Incubation of Cry1Ab activated toxin with a *Manduca sexta* cadherin fragment (CR7-12) triggered toxin oligomerization in solution^20^. Subsequent binding of the oligomeric Cry1A structure to ALP or APN facilitates its insertion into the membrane causing pore formation and cell lysis^17, 21^.

Recently, a novel insect molecule has been shown to be important in Cry1A toxicity since high levels of resistance to Cry1Ab or Cry1Ac are linked to different mutant alleles of an ABC transporter gene (ABCC2) in five lepidopteran insects, *Heliothis virescens*, *Plutella xylostella*, *Bombyx mori*, *Spodoptera exigua* and *Helicoverpa armigera* ^22–26^. It was also shown that mutations in another ABC transporter gene (ABCA2) was linked to Cry2Ab resistance in *H. armigera* ^27^. The role of ABC transporters in the mechanism of action of Cry toxins is not yet understood, although it was suggested that ABCC2 might be involved in facilitating Cry1A oligomer insertion into the membrane^28^. The mutation in ABCC2 in YEE strain of *H. virescens*, correlated with a loss of binding of Cry1Ab and Cry1Ac to brush border membrane vesicles (BBMV)^22^. Resistance to Cry1Ac in the *Tricoplusia ni* GlenBtR was shown to be closely linked to ABCC2 and was also affected in Cry1Ab and Cry1Ac binding to BBMV^23, 29^. However, no ABCC2 mutation was reported in the GlenBtR strain^23^. Interestingly, it was later reported that GlenBtR strain has a trans-acting effect that reduced the transcript levels of APN1 that is also a Cry1Ac receptor^30^. Different *P. xylostella* Cry1Ac resistant colonies have been characterized and most of them have been shown to share the same resistance mechanism linked to ABCC2 mutations as shown by complementation analysis^31^. However, a trans-acting factor was identified in *P. xylostella* as a MAPK component that is closely linked to ABCC2 and affects expression of different Cry1Ac receptors including ALP and ABCC2^32^. The *B. mori* ABCC2 transporter gene was cloned and expressed in SF9 cells and it was shown that it confers binding of Cry1A toxins and induces toxin susceptibility^33^. In addition, ABCC2 was identified as Cry1Ac binding molecule in *H. armigera* by pull-down assays strongly suggesting that ABCC2 is also a functional receptor of Cry1Ac toxin in this insect species^34^.

Some insects with mutations affecting their ABCC2 protein showed high resistance to Cry1Ab or Cry1Ac but were susceptible to the genetically modified toxins Cry1AbMod or Cry1AcMod^35–37^. These Cry1AMod toxins were engineered to have a deletion of the amino-terminal end of the toxin including helix α − 1^36^. These modified proteins do not require cadherin binding to form oligomeric structures *in vitro* and were able to overcome the high levels of resistance to native Cry1Ab or Cry1Ac toxins of *P. xylostella* strain NO-QAGE whose resistance is linked to an ABCC2 mutation^37^. To determine the role of ABCC2 in the Cry1Ac mode of action, we analyzed binding, oligomerization and insertion of toxin oligomer in BBMV isolated from *P. xylostella* susceptible to Cry1Ac and in the Cry1Ac-resistant strain NO-QAGE. We show that Cry1AcMod is able to counter resistance linked to ABCC2 due to its capacity to form oligomers that insert into the membrane of the NO-QAGE strain. Our data imply that ABCC2 is involved in inducing Cry1Ac oligomerization and in insertion of oligomer into the membrane.

## Results

### ABCC2 Alleles in Susceptible and Resistant Insects

When we amplified the 157-bp region from ABCC2 cDNA previously reported to contain the 30-bp deletion in the resistant NO-QAGE strain^23^, we obtained PCR products of the expected size and sequence from the NO-QAGE strain and the susceptible Geneva 88 strain (Fig. S1). Confirming the previous results, the previously reported 30-bp deletion occurred in the resistant strain but not in the susceptible strain (Fig. S1). Moreover, since no wild type PCR product was seen in the NO-QAGE cDNA sample, we concluded that the resistant strain was homogeneous.

### Binding of Cry1Ac and Cry1AcMod to BBMV from Resistant and Susceptible *P. xylostella*

To determine the effect of ABCC2 mutation in *P. xylostella* NO-QAGE strain on Cry1Ac and Cry1AcMod binding, we performed binding analysis of the activated toxins to BBMV prepared from the susceptible and resistant strains.

**Figure 1:**
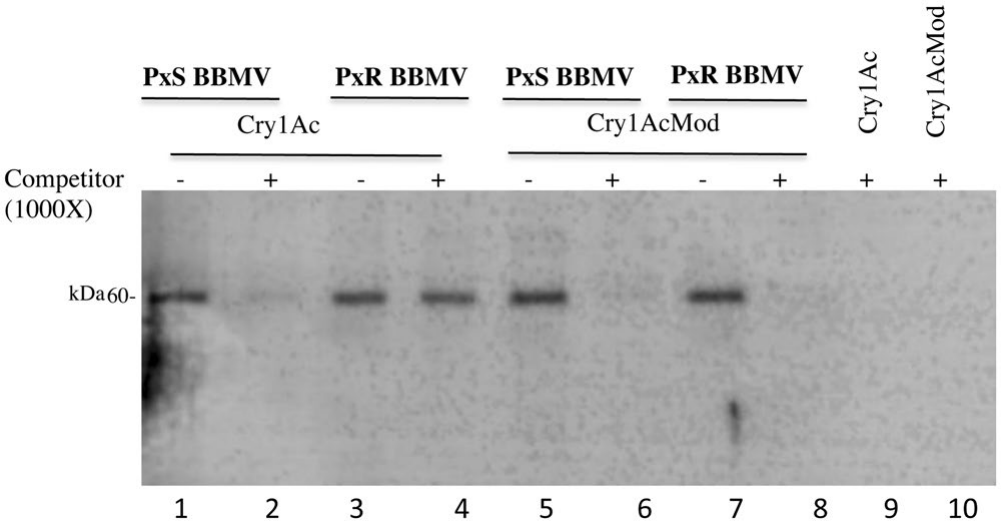
Total binding and non-specific binding of Cry1Ac and Cry1AcMod toxins to *Plutella xylostella*. Total binding and non-specific binding of biotinylated-Cry1Ac and Cry1AcMod toxins to BBMV isolated from *P. xylostella* susceptible Geneva 88 (PxS) and resistant NO-QAGE (PxR) populations. Lanes with negative symbol (−) represent total binding in absence of competitor and lanes with positive symbol (+) represent non-specific binding in the presence of 1000X unlabeled competitor. Lanes 1, 2, 3, and 4 are Cry1Ac labeled toxin incubated with BBMV from PxS (lanes 1 and 2) or PxR (lanes 3 and 4). Lanes 5, 6, 7 and 8 are Cry1AcMod labeled toxin incubated with BBMV from PxS (lanes 5 and 6) or PxR (lanes 7 and 8). Lane 9 and 10 are precipitation controls of labeled Cry1Ac (lane 9) or Cry1AcMod (lane 10) incubated with excess of unlabeled toxin without BBMV.

The crystal inclusions of Cry1Ac and Cry1AcMod were solubilized, activated with trypsin and labeled with biotin as described in Methods. The total binding of biotinylated proteins after incubation with BBMV isolated from each susceptible and resistant insect was analyzed in the absence of competitor. Total binding includes specific binding that corresponds to toxin bound to receptors and irreversible binding that corresponds to insertion of the toxin into the membrane. Specific binding was analyzed by determining the non-specific binding in homologous competition experiments after incubation of these toxins with the BBMV in the presence of 1000-fold excess of the corresponding unlabeled toxin. Figure 1 shows that Cry1Ac bound to BBMV from both susceptible Geneva 88 and resistant NO-QAGE strains. However, the binding of Cry1Ac to BBMV from NO-QAGE was non-specific since it was not competed by unlabeled Cry1Ac in contrast to the binding of Cry1Ac to BBMV from Geneva 88 (Fig. 1). Cry1AcMod bound BBMV from both strains and the binding was specific since it was competed by excess of unlabeled toxin.

### Oligomerization of Cry1Ac and Cry1AcMod Activated Toxins

**Figure 2:**
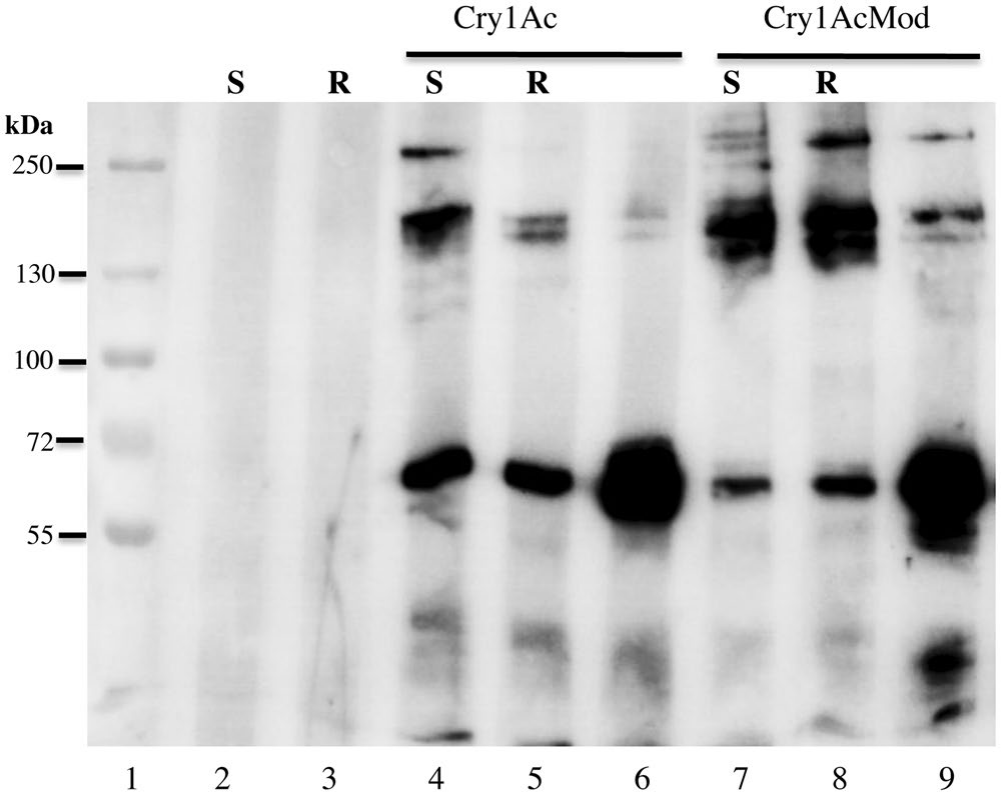
Oligomerization of Cry1Ac and Cry1AcMod in BBMV from susceptible and resistant populations of *P. xylostella*. Cry1Ac or Cry1AcMod activated toxins (1.5 μg) were incubated with BBMV (20 μg) from *P. xylostella* susceptible Geneva 88 or resistant NO-QAGE, BBMV were recovered by centrifugation and separated by SDS-PAGE after three min heating at 60 °C and revealed in western blot using anti-Cry1Ac antibody as described in Experimental procedures. Lane 1 shows Molecular weight marker; lane 2, BBMV from Geneva 88 without toxin; lane 3, BBMV from NO-QAGE without toxin; lane 4, BBMV from Geneva 88 with Cry1Ac toxin; lane 5, BBMV from NO-QAGE with Cry1Ac toxin; lane 6 is a control of activated Cry1Ac toxin directly loaded in the SDS-PAGE; lane 7, BBMV from Geneva 88 with Cry1AcMod toxin; lane 8, BBMV from NO-QAGE with Cry1AcMod toxin and lane 9 is a control of activated Cry1AcMod toxin directly loaded in the SDS-PAGE.

We analyzed oligomerization of Cry1Ac and Cry1AcMod after incubation of activated toxins with BBMV separated by centrifugation from the susceptible and resistant insect strains using western blots (Fig. 2). This assay determines the oligomer that is associated with the BBMV that is likely to be inserted into the membrane and may be responsible for pore formation. For Cry1Ac, the optical density analysis of the 200 kDa bands indicated that oligomer formation was significantly greater with BBMV from the susceptible strain than with BBMV from the resistant strain (Table S1, paired t-test, t = 8.6, df = 2, P = 0.01). By contrast, for Cry1AcMod, the optical density of the 200 kDa bands indicated that oligomer formation did not differ significantly between BBMV from the susceptible strain and the resistant strain (Table S1, paired t-test, t = 0.02, df = 2, P = 0.98). Relative to the optical density of the 200 kDa bands formed with BBMV from the susceptible strain (=100%), the mean optical density of 200 kDa bands formed with BBMV from the resistant strain was 31.6% (SE = 11.7%) for Cry1Ac and 100.2% (SE = 7.4%) for Cry1AcMod.

**Figure 3:**
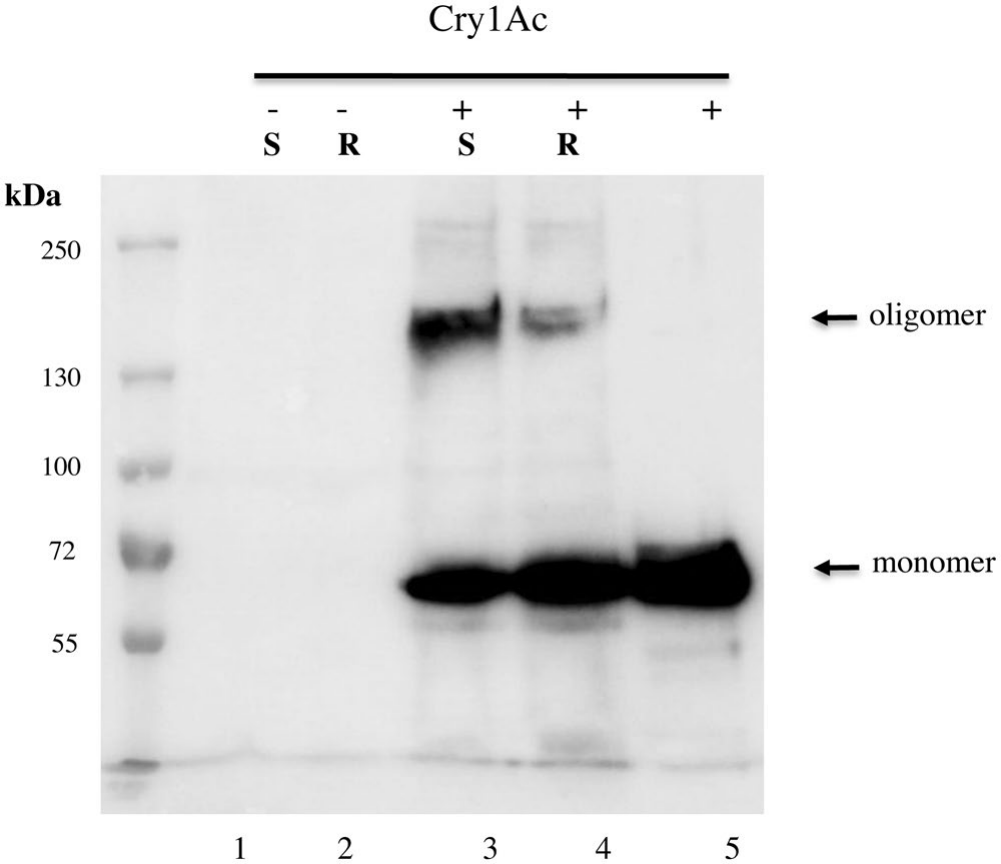
Oligomerization of Cry1Ac in the presence of BBMV from susceptible and resistant populations of *P. xylostella*. Cry1Ac activated toxin (1.5 μg) was incubated with BBMV (20 μg) from *P. xylostella* susceptible Geneva 88 or resistant NO-QAGE, the whole sample was separated by SDS-PAGE after three min heating at 60 °C and revealed in western blot using anti-Cry1Ac antibody as described in Experimental procedures. Lane 1, BBMV from Geneva 88; lane 2, BBMV from NO-QAGE; lane 3, Cry1Ac incubated with BBMV from Geneva 88; lane 4, Cry1Ac incubated with BBMV from NO-QAGE; lane 5, Cry1Ac toxin without BBMV.

To analyze total oligomer formation, BBMV were not separated by centrifugation after incubation with Cry1Ac (Fig. 3). This assay determines the total oligomer formation regardless if it is associated with the membrane or not. The optical density analysis of the 200 kDa bands indicated that oligomer formation of Cry1Ac was significantly greater with BBMV from the susceptible strain than with BBMV from the resistant strain (Fig. 3, Table S1, paired t-test, t = 6.4, df = 2, P = 0.02).

### Insertion of Pre-formed Cry1Ac Oligomer in BBMV from Both Strains

**Figure 4:**
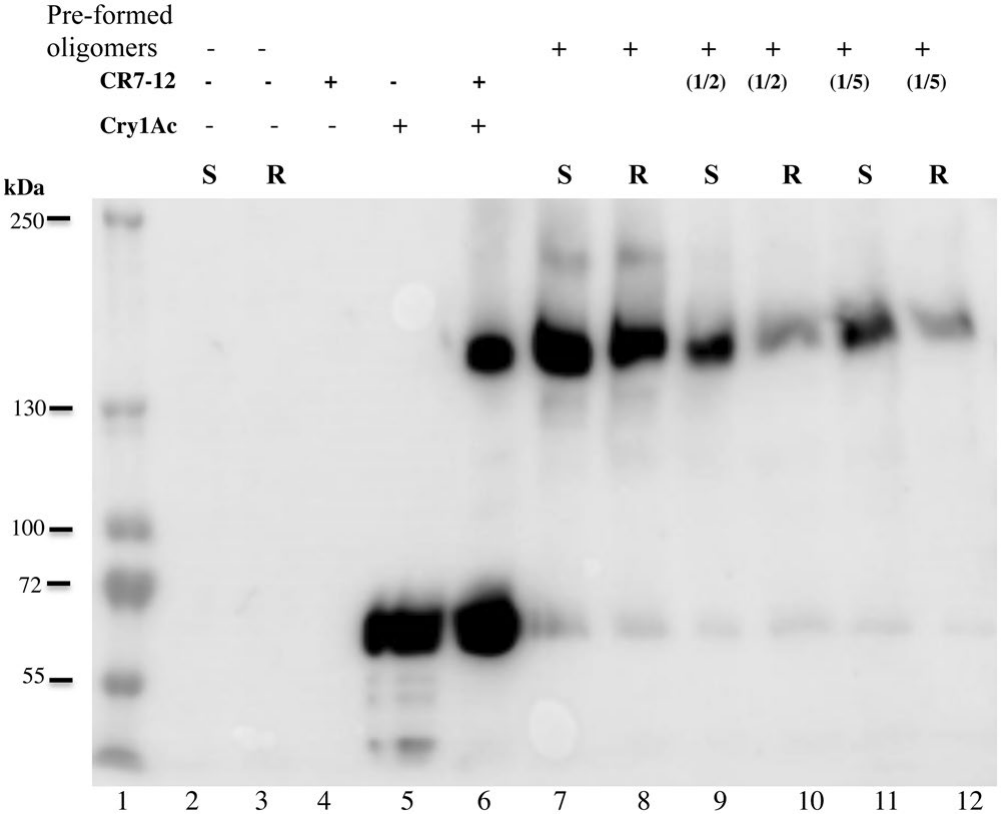
Association of preformed Cry1Ac oligomer with BBMV from susceptible and resistant populations of *P. xylostella*. One μg of Cry1Ac toxin was incubated with *M. sexta* cadherin fragment CR7-CR12 (1:4) for two h and Cry1Ac oligomers were revealed by western blot as described in experimental procedures. BBMV (20 μg) were incubated 1 h with pre-formed oligomers and BBMV were recovered by centrifugation and revealed by western blot. Lane 1, molecular weight markers; lane 2, BBMV from Geneva 88; lane 3, BBMV from NO-QAGE; lane 4, CR7-CR12 protein fragment; lane 5, Cry1Ac activated toxin; lane 6, CR7-CR12 fragment with Cry1Ac toxin; lane 7, BBMV from Geneva 88 incubated with preformed Cry1Ac oligomer; lane 8, BBMV from NO-QAGE incubated with preformed Cry1Ac oligomer; lane 9, BBMV from Geneva 88 incubated with preformed Cry1Ac oligomer diluted two-fold; lane 10, BBMV form NO-QAGE incubated with preformed Cry1Ac oligomer diluted two-fold; lane 11, BBMV from Geneva 88 incubated with preformed Cry1Ac oligomer diluted five-fold; lane 12, BBMV from NO-QAGE incubated with preformed Cry1Ac oligomer diluted five-fold.

The lower amounts of Cry1Ac oligomers in BBMV membranes from NO-QAGE could be consistent with a defect in oligomer membrane insertion. In order to find out if ABCC2 was involved in promoting insertion of oligomeric Cry1Ac into the membrane, we analyzed the association of pre-formed oligomers with BBMV from both Geneva 88 and NO-QAGE strains as described in Methods. This assay determines the capacity of soluble Cry1Ac oligomers to associate with the BBMV that are likely to be inserted into the membrane. When undiluted pre-formed oligomers of Cry1Ac were incubated with BBMV to test for membrane insertion, the optical density of 200 kDa bands indicated that oligomers were slightly, but not significantly lower with BBMV from the resistant strain relative to BBMV from the susceptible strain (Fig. 4, Table S1, paired t-test, t = 1.3, df = 2, P = 0.33). By contrast, the optical density of 200 kDa bands was significantly greater for BBMV from the susceptible strain than for BBMV from the resistant strain when pre-formed oligomers were diluted by half or one fifth (Fig. 4, Table S1, paired t-tests, df = 2 for each test; for half t = 4.7, P = 0.04 and for one fifth t = 12.6, P = 0.006). Relative to the optical density of 200 kDa bands formed with BBMV from the susceptible strain (=100%), the mean optical density of 200 kDa bands formed with BBMV from the resistant strain was 88.5% (SE = 16.4%) for the undiluted pre-formed oligomers of Cry1Ac, 56.0% (SE = 17.4%) for the pre-formed oligomers diluted by one half, and 28.2% (SE = 18.6%) for the pre-formed oligomers diluted by one fifth.

## Discussion

The role of ABCC2 in the mode of action of Cry1A toxins has not been clear. Characterization of the binding of Cry1Ab or Cry1Ac in resistant colonies from different lepidopteran species suggested that ABCC2 might function as receptor molecule since in most cases at least one Cry1A toxin is affected in binding to BBMV from the resistant colony^22, 29^. To gain insight into the possible role of ABCC2 in the mode of action of Cry1Ac toxin we compared the binding and oligomerization of Cry1Ac and Cry1AcMod toxins to BBMV from a strain﻿ of *P. xylostella* susceptible to Cry1Ac and a strain in which resistance to Cry1Ac is linked to an ABCC2 mutation^23^.

We first confirmed the ABCC2 allele mutation in NO-QAGE strain used in this study by amplifying an ABCC2 gene fragment containing the reported mutation^23^. We confirmed the 30 bp deletion in NO-QAGE strain. Moreover, since the RNA samples were obtained by pooling 20 larvae and no wt PCR product was observed using the cDNA from NO-QAGE sample, we conclude that the Cry1Ac- selected NO-QAGE strain was homogenous.

Binding analysis showed that Cry1Ac and Cry1AcMod toxins bound *P. xylostella* Geneva 88 and NO-QAGE BBMV (Fig. 1). However, binding of Cry1Ac to BBMV from NO-QAGE was not specific since it was not competed by an excess of unlabeled toxin (Fig. 1). Previous binding analysis using iodide-labeled Cry1Ac showed reduced binding of this toxin to BBMV from the NO-QA strain^38, 39^. NO-QAGE was created by crossing NO-QA, a field-selected resistant strain from Hawaii with the Geneva 88 strain, followed by selection of the F3 progeny with Cry1Ac^40, 41^. We observed that specific binding of Cry1Ac to NO-QAGE BBMV was reduced, but total binding was not, which differs from previous reports that total binding of Cry1Ac was diminished in the NO-QA BBMV^38, 39^. This difference could reflect differences between NO-QA and NO-QAGE, differences in methods between studies, or both. In *S. exigua*, Cry1Ca and Cry1Ac resistance was linked to a deletion in ABCC2 corresponding to one of the ATP-binding domains^25^. Analysis of binding revealed that Cry1Ca total binding to BBMV was not significantly reduced in the resistant Xen-r strain but most of the binding was not specific since it was only partially competed by unlabelled toxin^25^. These data are similar to what we observed in the NO-QAGE BBMV. Our results show that resistance of NO-QAGE to Cry1Ac correlates with reduced specific binding and that the Cry1AcMod toxin that is toxic to NO-QAGE recovered specific binding to NO-QAGE BBMV. One would expect that non-specific binding of Cry1Ac should be similar between susceptible and resistant strains. It could be possible that the lack of ABCC2 in membranes could be compensated by increased expression of other membrane proteins or membrane lipids that contribute to non-specific binding of the toxin.

We report here that the oligomerization of Cry1Ac is reduced in BBMV from Cry1Ac-resistant NO-QAGE that correlates also with resistance to this toxin (Fig. 2). We have previously shown that irreversible binding of Cry1Ab toxin to *M. sexta* BBMV is due to oligomer membrane insertion^20^. By analyzing the amount of oligomers formed in samples where BBMV were not separated by centrifugation we showed that less oligomers are formed in BBMV from NO-QAGE (Fig. 3) indicating that ABCC2 is involved in facilitating oligomerization of Cry1Ac toxin. However, we cannot exclude the possibility that reduced specific binding in the NO-QAGE BBMV causes reduced oligomer formation in NO-QAGE BBMV. Here we show that Cry1AcMod formed oligomers that associate with BBMV from both the susceptible Geneva 88 and the Cry1Ac resistant NO-QAGE explaining its capacity to overcome Cry1Ac-resistance^37^. However, it was previously reported that an *H. armigera* Cry1Ac resistant colony linked to an ABCC2 mutation was not affected in Cry1Ac oligomerization^42^. The LF120 strain, where oligomerization assays were performed^42^, was derived from LF60^26^ by further selection with Cry1Ac and shown to contain the same ABCC2 mutant allele as LF60 (Kongming Wu personal communication). The ABCC2 allele in LF60 showed a 73 bp DNA insertion that introduced a premature stop codon resulting in ABCC2 protein lacking 143 amino acids from the c-terminal end ref. 26. We do not know the reason for this different result, it is possible that ABCC2 has a different role in Cry1A toxin oligomerization in different insect species. Also, differences in the oligomerization assays may account for this discrepancy, since the analysis of oligormerization of Cry1Ac in *H. armigera* was done using Cry1Ac protoxin and here we analyzed the oligomerization of activated Cry1Ac toxin. Finally, differences in oligomerization could be due to the differences in the ABCC2 mutant alleles between the two insect strains. We have previously shown that two different oligomeric structures could be formed depending if protoxin or activated toxins interact with the insect BBMV^20^.

It was proposed that ABCC2 functions as a receptor molecule that binds the oligomeric structure and facilitates its insertion into the membrane^28^. To find out if ABCC2 could also be involved in such step, we analyzed the association of pre-formed Cry1Ac oligomers in solution with BBMV from both Geneva 88 and NO-QAGE. Figure 4 shows that very similar amounts of Cry1Ac oligomers were associated with BBMV when the oligomer sample was not diluted and incubated with BBMV from both susceptible and Cry1Ac-resistant strains. However, when we performed the association of pre-formed oligomers using two serial dilutions of the original Cry1Ac oligomer sample to perform the analysis in non-saturated conditions, less Cry1Ac oligomers were associated with BBMV from NO-QAGE in comparison with BBMV from the susceptible Geneva-88 strain (Fig. 4). These results indicate that ABCC2 is partially involved in facilitating oligomer membrane insertion. It could be possible that other membrane associated molecules such as ALP or APN in BBMV could still favor oligomer membrane insertion explaining the partial effect of ABCC2 mutation in oligomer association with BBMV. Previous data suggested that binding to *M. sexta* APN or ALP facilitates the membrane insertion of Cry1Ac or Cry1Ab oligomers^17, 21^.

Recently, we reported that Cry1Ac deficiency in oligomerization in BBMV from *Pectinophora gossypiella* correlated with Cry1Ac-resistance linked to cadherin mutations and that Cry1AcMod was still able to form oligomers explaining its capacity to counter resistance^43^. Our results show a very similar phenotype for the *P. xylostella* ABCC2 mutant since lower oligomerization was observed in the resistant strain (Fig. 2) suggesting that ABCC2 and cadherin have a similar role in Cry1Ac oligomerization in different insect species. Our data also supports that ABCC2 is not only involved in facilitating oligomerization of Cry1Ac toxin but also in oligomer membrane insertion (Figs 3 and 4). These data agree with the fact that Cry1AcMod counters resistance to Cry1Ac in the NO-QAGE ABCC2 mutant since Cry1AcMod still forms oligomers in the absence of receptor binding^36, 37^. Interestingly, it was shown that Cry1Aa binds *B. mori* cadherin or ABCC2 by means of the same domain II loop regions supporting a similar role of both receptor molecules in Cry1Aa toxin action^44^. It is possible that both ABCC2 and cadherin participate together in efficient Cry1A toxin oligomerization explaining why oligomerization was affected but not abolished in Cry1Ac resistant *P. gossypiella* linked to cadherin mutations^43^ as was also the case for Cry1Ac resistant *P. xylostella* linked to ABCC2 mutation (Fig. 2). NO-QAGE still produces cadherin as shown by western blot analysis (data not shown). In a strain of *H. virescens* with both mutant cadherin and ABCC2, resistance was higher than in strains with only one of the two mutations^22^. However, in *H. virescens* ABBC2 seems to play a major role based on the higher resistance levels observed in the resistant strain containing only the ABCC2 mutations compared with the single cadherin mutation^22^. In *B. mori*, the expression of ABCC2 or cadherin genes in SF9 cells showed that ABCC2 expression conferred 1000-fold greater susceptibility to Cry1Aa toxin than cadherin expression, suggesting also that ABCC2 might be more important for toxicity in this insect species^33^. The higher resistance levels observed in the *H. virescens* ABCC2 mutant in comparison with the cadherin mutant and the higher susceptibility to Cry1Aa of SF9 cells expressing *B. mori* ABCC2 in comparison with cadherin expressing cells, could be explained since ABCC2 is involved in Cry1Ac toxin oligomerization and oligomer membrane insertion in contrast to cadherin that is involved only in facilitating oligomerization of the toxin. Interestingly, the expression of both cadherin and ABCC2 from *B. mori* in SF9 cells revealed a synergism between cadherin and ABCC2 since SF9 cells expressing both receptor molecules were far more sensitive to Cry1Aa than cells expressing only ABCC2^33^. Expression of ABCC2 and cadherin (HevCALP) from *H. virescens* in SF9 cells also revealed a synergistic interaction of both receptors in Cry1Ac toxicity^45^. It was proposed that HevCALP might serve as a sink of membrane-inserted oligomers facilitating the role of ABCC2 in insertion of additional pre-pore oligomers into the membrane^45^. It was recently shown that expression of *B. mori* ABBC2 in frog oocytes was sufficient to trigger pore formation, suggesting that ABCC2 is involved in toxin oligomerization and in membrane insertion and that co-expression of ABCC2 with cadherin greatly enhanced Cry1Aa pore formation supporting also a role of cadherin in assisting ABCC2 in triggering oligomer membrane insertion and pore formation^46^. In addition, Cry1Aa oligomers were observed in similar amounts in oocytes expressing either cadherin or ABCC2 indicating that both cadherin and ABCC2 trigger Cry1Aa oligomerization but only ABCC2 facilitates efficient oligomer membrane insertion and pore formation^46^. Our data show that in *P. xylostella*, ABCC2 is involved in efficient oligomerization and membrane insertion of activated toxin and that resistance correlates with reduced oligomerization of Cry1Ac in the target insect membranes.

## Methods

### Insect Strains and ABCC2 Allele Confirmation

The Cry1Ac resistant strain (NO-QAGE) and the susceptible strain (Geneva 88) from *P. xylostella* were purchased from Benzon Research Inc. (www.benzonresearch.com). NO-QAGE population was selected with Cry1Ac toxin. Geneva 88 strain was originated in 1988 from a cabbage field near Geneva, New York^47^. NO-QAGE was created by crossing NO-QA, a field-selected resistant strain from Hawaii^40^, with the susceptible strain Geneva 88, followed by selection of the F3 progeny in the presence of Cry1Ac toxin^41^. It was previously reported that the Cry1Ac resistance of NO-QAGE strain was linked to an ABCC2 mutation that involved a deletion of ten amino acids, eliminating the transmembrane helix 12 of the transporter^23^. To verify the ABBC2 mutant allele in the NO-QAGE population characterized in this work, we amplified a 157 bp region that contains the ABCC2 mutation from RNA samples of both sensitive Geneva 88 and resistant NO-QAGE strains. Twenty independent larvae of each strain, reared without Cry1Ac toxin, were pooled to obtain RNA samples and cDNA was synthesized., Total RNA from twenty 4^th^ instar midguts of *P. xylostella* strains Geneva 88 or NO-QAGE was extracted using RNeasy Mini kit (Qiagen). cDNA was synthesized from 1 μg total RNA using SuperScript^TM^ III Reverse Transcriptase kit (Invitrogen Life Technologies), following the manufacturer’s instructions. ABCC2 exon 20 gene region of 157 pb was amplified by PCR using the following primers: PxDelFwd 5′-gat atg cct cgt cta cct cgc-3′and PxDelRev 5′-cag gaa gtc gct ggt gaa c-3′. The amplification conditions were 5 min at 95 °C followed by 30 cycles of 30 s at 95 °C, 30 s at 60 °C and 30 s at 72 °C with a final extension step of 10 min at 72 °C using Taq DNA polymerase (Altaenzymes). The PCR products sizes were analyzed by agarose (2%) gel electrophoresis and DNA sequence was obtained using primer PxDelFwd at the DNA sequencing facilities of IBT, UNAM.

### Cry1Ac and Cry1AcMod Toxin Purification

Bt HD73 expressing Cry1Ac or Bt 407 expressing Cry1AcMod^36^ were grown at 30 °C until complete sporulation (3 to 4 days) in nutrient broth sporulation medium. In the case of Cry1AcMod the medium was supplemented with erythromycin at 10 μg ml^−1^. Spores/crystals were washed twice in 300 mM NaCl, 10 mM EDTA. Crystal inclusions were solubilized in an alkaline buffer (50 mM Na_2_CO_3_, 0.2% β-mercaptoethanol, pH 10.5) for 2 h at 37 °C. Trypsin activated toxins were obtained by treatment of soluble protoxin with trypsin (TPCK treated trypsin from bovine pancreas, SIGMA Aldrich, St. Louis, MO) in a mass ratio of 1:50 (trypsin: toxin) for 2 h at 37 °C after lowering the pH to 8.5 by adding 1:4 (w/w) of 1 M Tris buffer pH 8.5. Phenylmethylsulfonyl fluoride (PMSF) (1 mM final concentration) was added to stop proteolysis. Activated proteins were purified by anion exchange chromatography Mono Q-Sepharose fast flow (GE Healthcare, Little Chalfont, UK) in an AKTA FPLC System (GE Healthcare, Little Chalfont, UK), using a 50 mM Tris-HCl, 50 mM NaCl, pH 8.5 buffer, and a linear NaCl concentration gradient from 50 to 300 mM. Protein concentrations were determined by the method of Bradford, using bovine serum albumin as a standard.

### Midgut Brush Border Membrane Vesicles (BBMV) Purification

*P. xylostella* midgut tissues from 3^rd^ instar larvae were dissected and stored immediately at −70 °C. BBMV were prepared by the magnesium precipitation method without protease inhibitors as described by Wolfersberger 1993^48^ and stored at −70 °C until used. The BBMV protein concentrations were determined with the Lowry DC protein assay (BioRad, Hercules, CA) using bovine serum albumin as a standard.

### Binding of Cry1Ac and Cry1AcMod to BBMV

Trypsin activated monomeric toxins were labeled with biotinyl-*N-*hydroxy-succinimide ester according to the manufacturer’s instructions (Amersham Biosciences). Binding of labeled toxins was analyzed by incubating 5 nM labeled toxin with 10 μg BBMV protein for 100 min at 25 °C in 100 μl binding buffer (PBS, 0.1% BSA, 0.1% Tween 20, pH 7.6). Non-specific binding was determined by measuring binding of 5 nM labeled toxin in the presence of 1000-fold molar excess of unlabeled toxin after 100 min. After incubation, the unbound toxin was removed by centrifugation for 10 min at 14,000 x*g*. The pellet containing BBMV and bound toxin was washed twice with 100 μl binding buffer, suspended in 10 μl of PBS pH 7.6, and 10 μl sample loading Laemmli buffer 2X (0.125 mM Tris-HCl, pH 6.8, 4% SDS, 20% glycerol, 10% 2-mercaptoethanol, and 0.01% bromophenol blue). Samples were boiled 3 min, loaded in 10% SDS-PAGE gels and electrotransferred to nitrocellulose membranes. Bound labeled toxin was identified by incubating with streptavidin-peroxidase conjugate (Millipore) (1:20000 dilution) for 1 h and developed with luminol (Santa Cruz Biotechnology Inc.). Binding assays were performed in triplicate.

### Toxin Oligomerization in BBMV and in Solution

Oligomerization of Cry1Ac or Cry1AcMod toxins in BBMV was analyzed as previously described^22^. Activated toxins, 1.5 μg, were incubated with 20 μg of BBMV protein for 1 h at 37 °C in a total volume of 50 μl of 50 mM Na_2_CO_3_, pH 10.5. Control samples contained only BBMV. The reactions were stopped with 1 mM PMSF and the BBMV were recovered by 30 min centrifugation at 50,000 rpm at 4 °C. The pellet was washed once with 100 μl of 50 mM Na_2_CO_3_, and finally suspended in 50 μl of the same buffer. Laemmli sample buffer 4X was added and incubated three min at 60 °C. We also analyzed some samples where the BBMV were not separated by centrifugation, Laemmli sample buffer 4X was added and incubated three min at 60 °C. After heating, samples were separated in 8% SDS-PAGE, electro transferred to PVDF membrane and revealed in western blot assays using anti-Cry1Ac antibody (1/30,000; 1 h) as the primary antibody. As secondary antibody, a goat anti-rabbit secondary antibody coupled to horseradish peroxidase was used (Santa Cruz Biotechnology, Dallas, TX) (1/25,000; 1 h), followed by luminol (Santa Cruz Biotechnology Inc.), according to the manufacturer’s instructions. For oligomer formation in solution, 1 μg of Cry1Ac activated toxin was incubated with the cadherin fragment CR7-CR12 containing residues 810–1480 in a mass ratio of 1:4 (toxin:CR7-CR12) for 2 h and analyzed by western blot as described above. CR7-CR12 was expressed in *E. coli* ER2566 and purified using nickel affinity as previously described^49^. To analyze oligomer association with BBMV, 20 μg of BBMV from susceptible or resistant populations were added to undiluted Cry1Ac-oligomer samples or Cry1Ac-oligomer samples that were diluted two or five-fold and incubated for 1 h. Membrane pellets were recovered by centrifugation and analyzed by western blot as described above. All assay were performed in triplicate. The MW of the 200 kDa oligomer was established by using a pre-stained MW marker. (PageRuler Plus prestained, Thermo Scientific). The optical density of the 200 kDa bands was measured by using ImageJ program (http://imagej.nih.gov/ij/).

### Data availability

All data generated or analyzed during this study are included in this published article (and its Supplementary Information files).

## Electronic supplementary material

Supplementary Information

## References

[1] Bravo A, Likitvivatanavong S, Gill SS, Soberón M (2011). *Bacillus thuringiensis*: a story of a successful bioinsecticide. Insect Biochem. Mol. Biol..

[2] Sanahuja G, Banakar R, Twyman RM, Capell T, Christou P (2011). *Bacillus thuringiensis*: a century of research development and commercial applications. Plant Biotechnol. J..

[3] Tabashnik BE, Brévault T, Carrière Y (2013). Insect resistance to Bt crops: lessons from the first billion acres. Nat. Biotechnol..

[4] Carrière Y, Fabrick JA, Tabashnik BE (2016). Can pyramids and seed mixtures delay resistance to Bt crops?. Trends Biotechnol..

[5] Gassmann AJ (2014). Field-evolved resistance by western corn rootworm to multiple *Bacillus thuringiensis* toxins in transgenic maize. Proc. Natl. Acad. Sci. USA.

[6] Jakka SRK, Shrestha RB, Gassmann AJ (2016). Broad-spectrum resistance to Bacillus thuringiensis toxins by western corn rootworm (*Diabrotica virgifera virgifera*). Sci. Rep..

[7] Dively GP, Venugopal PD, Finkenblnder C (2016). Field-evolved resistance in corn earworm to Cry proteins expressed by transgenic sweet corn. Plos One..

[8] Farias JR (2014). Field-evolved resistance to Cry1F maize by *Spodoptera frugiperda* (Lepiodptera: Noctuidae) in Brazil. Crop. Prot..

[9] Monnerat R (2015). Evidence of field-evolved resistance of *Spodoptera frugiperda* to Bt corn expressing Cry1F in Brazil that is still sensitive to modified Bt toxins. PLoS ONE.

[10] Ferré J, Van Rie J (2002). Biochemistry and genetics of insect resistance to *Bacillus thuringiensis*. Annu. Rev. Entomol..

[11] Wu Y (2014). Detection and mechanisms of resistance evolved in insects to Cry toxins from *Bacillus thuringiensis*. Adv. Insect Physiol..

[12] Soberón M, Gill SS, Bravo A (2009). Signaling versus punching hole: How do *Bacillus thuringiensis* toxins kill insect midgut cells?. Cell. Mol. Life. Sci..

[13] Bravo A (2004). Oligomerization triggers binding of a *Bacillus thuringiensis* Cry1Ab pore-forming toxin to aminopeptidase N receptor leading to insertion into membrane microdomains. Biochim. Biophys. Acta..

[14] Pigott CR, Ellar DJ (2007). Role of receptors in *Bacillus thuringiensis* crystal toxin activity. Microbiol. Mol. Biol. Rev..

[15] Pacheco S (2009). Domain II loop 3 of *Bacillus thuringiensis* Cry1Ab toxin is involved in a “ping-pong” binding mechanism with *Manduca sexta* aminopetidase-N and cadherin receptors. J. Biol. Chem..

[16] Masson L, Lu YJ, Mazza A, Brousseau R, Adang MJ (1995). The Cry1A(c) receptor purified from *Manduca sexta* displays multiple specificities. J. Biol. Chem..

[17] Arenas I, Bravo A, Soberón M, Gómez I (2010). Role of alkaline phosphatase from *Manduca sexta* in the mechanism of action of *Bacillus thuringiensis* Cry1Ab toxin. J. Biol. Chem..

[18] Upadhyay SK, Singh PK (2011). Role of alkaline phosphatase in insecticidal action of Cry1Ac against *Helicoverpa armigera* larvae. Biotechnol. Lett..

[19] Gómez I, Sánchez J, Miranda R, Bravo A, Soberón M (2002). Cadherin-like receptor binding facilitates proteolytic cleavage of helix α-1 in domain I and oligomer pre-pore formation of *Bacillus thuringiensis* Cry1Ab toxin. FEBS. Lett..

[20] Gómez I (2014). *Bacillus thuringiensis* Cry1A toxins are versatile-proteins with multiple modes of action: Two distinct pre-pores are involved in toxicity. Biochem. J..

[21] Pardo-López L (2006). Structural changes of the Cry1Ac oligomeric pre-pore from *Bacillus thuringiensis* induced by *N*-acetylgalactosamine facilitates toxin membrane insertion. Biochem..

[22] Gahan LJ, Pauchet Y, Vogel H, Heckel DG (2010). An ABC transporter mutation is correlated with insect resistance to *Bacillus thuringiensis* Cry1Ac toxin. PLoS Genet..

[23] Baxter SW (2011). Parallel evolution of Bt toxin resistance in Lepidoptera. Genetics..

[24] Atsumi S (2012). Single amino acid mutation in an ATP-binding cassette transporter causes resistance to Bt toxin Cry1Ab in the silkworm. Bombyx mori. Proc. Natl. Acad. Sci. USA.

[25] Park Y (2014). ABCC transporters mediate insect resistance to multiple Bt toxins revealed by bulk segregant analysis. BMC Biol..

[26] Xiao Y (2014). Mis-splicing of the ABCC2 gene linked with Bt toxin resistance in *Helicoverpa armigera*. Sci. Rep..

[27] Tay WT (2015). Insect resistance to *Bacillus thuringiensis* toxin Cry2Ab is conferred by mutations in an ABC transporter subfamily A protein. PLoS Genet..

[28] Heckel DG (2012). Learning the ABCs of Bt: ABC transporters and insect resistance to *Bacillus thuringiensis* provide clues to a crucial step in toxin mode of action. Pest. Biochem. Physiol..

[29] Wang P (2007). Mechanism of resistance to *Bacillus thuringiensis* toxin Cry1Ac in a greenhouse population of the cabbage looper, *Trichoplusia ni*. Appl. Environ. Microbiol..

[30] Tiewsiri K, Wang P (2011). Differential alteration of two aminopeptidases N associated with resistance to *Bacillus thuringiensis* toxin Cry1Ac in cabbage looper. Proc. Natl. Acad. Sci. USA..

[31] Crickmore N (2016). *Bacillus thuringiensis* resistance in Plutella- too many trees. Curr. Op. Ins. Sci.

[32] Guo Z (2015). MAPK signaling pathway alters expression of midgut ALP and ABCC genes and causes resistance to *Bacillus thuringiensis* Cry1Ac toxin in diamonback moth. PLoS Genet..

[33] Tanaka S (2013). The ATP-binding cassette transporter subfamily C member 2 in *Bombyx mori* larvae is a functional receptor for Cry toxins from *Bacillus thuringiensis*. FEBS J..

[34] Zhou Z (2016). Identification of ABCC2 as a binding protein of Cry1Ac on brush border membrane vesicles from *Helicoverpa armigera* by an improved pull-down assay. Microbiol. Open.

[35] Franklin MT (2009). Modified *Bacillus thuringiensis* toxins and a hybrid *B. thuringiensis* strain counter greenhouse-selected resistance in *Trichoplusia ni*. Appl. Environ. Microbiol..

[36] Soberón M (2007). Engineering Modified Bt Toxins to Counter Insect Resistance. Scie.

[37] Tabashnik BE (2011). Efficacy of genetically modified Bt toxins against insects with different mechanisms of resistance. Nat. Biotechnol..

[38] Tabashnik, B. E. *et al*. Reversal resistance to *Bacillus thuringiensis* in *Plutella xylostella*. *Proc. Natl. Acad. Sci. USA*. **91**, 4120–4124 (1994). 10.1073/pnas.91.10.4120 PMC43736 8183881

[39] Tabashnik BE (1997). Global variantion in the genetic and biochemical basis of diamondback moth resistance to *Bacillus thuringiensis*. Proc. Natl. Acad. Sci. USA..

[40] Tabashnik BE, Liu YB, Finson N, Masson L, Heckel DG (1997). One gene in diamondback moth confers resistance to four *Bacillus thuringiensis* toxins. Proc. Natl. Acad. Sci. USA..

[41] Tabashnik BE, Johnson KW, Engleman JT, Baum JA (2000). Cross-resistance to *Bacillus thuringiensis* toxin Cry1Ja in a strain of diamondback moth adapted to artificial diet. J. Invertebr. Pathol..

[42] Li Y (2012). Comparative analysis of Cry1Ac toxin oligomerization and pore formation between Bt-susceptible and Bt-resistant *Helicoverpa armigera* larvae. J. Integr. Agricul.

[43] Ocelotl J (2015). Binding and oligomerization of modified and native Bt toxins in resistant and susceptible Pink Bollworm. PLoS ONE..

[44] Adegawa S (2016). The domain II loops of *Bacillus thuringiensis* Cry1Aa form and overlapping interaction site for two *Bombyx mori* larvae functional receptors, ABC transporter C2 and Cadherin-like receptor. BBA-Proteins Proteom.

[45] Bretschneider, A., Heckel, D. G. & Pauchet, Y. Three toxins, two receptors, one mechanism: Mode of action of Cry1A toxins from *Bacillus thrungiensis* in *Heliothis virescens*. *Insect Biochem. Mol. Biol*. **76**, 109–117 (2016). 10.1016/j.ibmb.2016.07.008 27456115

[46] Tanaka S, Endo H, Adegawa S, Kikuta S, Sato R (2016). Functional characterization of *Bacillus thuringiensis* Cry toxin receptors explain resistance in insects. FEBS J..

[47] Shelton AM (1993). Resistance of diamondback moth (Lepidoptera: Plutellidae) to *Bacillus thuringiensis* subspecies in the field. J. Econom. Entomol..

[48] Wolfersberger MG (1993). Preparation and partial characterization of amino acid transporting brush border membrane vesicles from the larval midgut of the gypsy moth (*Lymantria dispar*). Arch, Insect Biochem. Physiol..

[49] Jiménez-Juárez N (2007). *Bacillus thuringiensis* Cry1Ab mutants affecting oligomer formation are non toxic to *Manduca sexta* larvae. J. Biol. Chem..

