# Supplementary material for: ABCC2 is associated with Bacillus thuringiensis Cry1Ac toxin oligomerization and membrane insertion in diamondback moth
Source: Sci Rep. 2017 May 24;7:2386. doi: 10.1038/s41598-017-02545-y (PMC5443830; doi:10.1038/s41598-017-02545-y)
Supplement: Supplementary file 1 — Supplementary Information [file 41598_2017_2545_MOESM1_ESM.pdf]

ABCC2 is associated with *Bacillus thuringiensis* Cry1Ac toxin oligomerization and membrane insertion in diamondback moth

Josue Ocelotl, Jorge Sánchez, Isabel Gómez, Bruce E. Tabashnik, Alejandra Bravo and Mario Soberón*

Fig S1

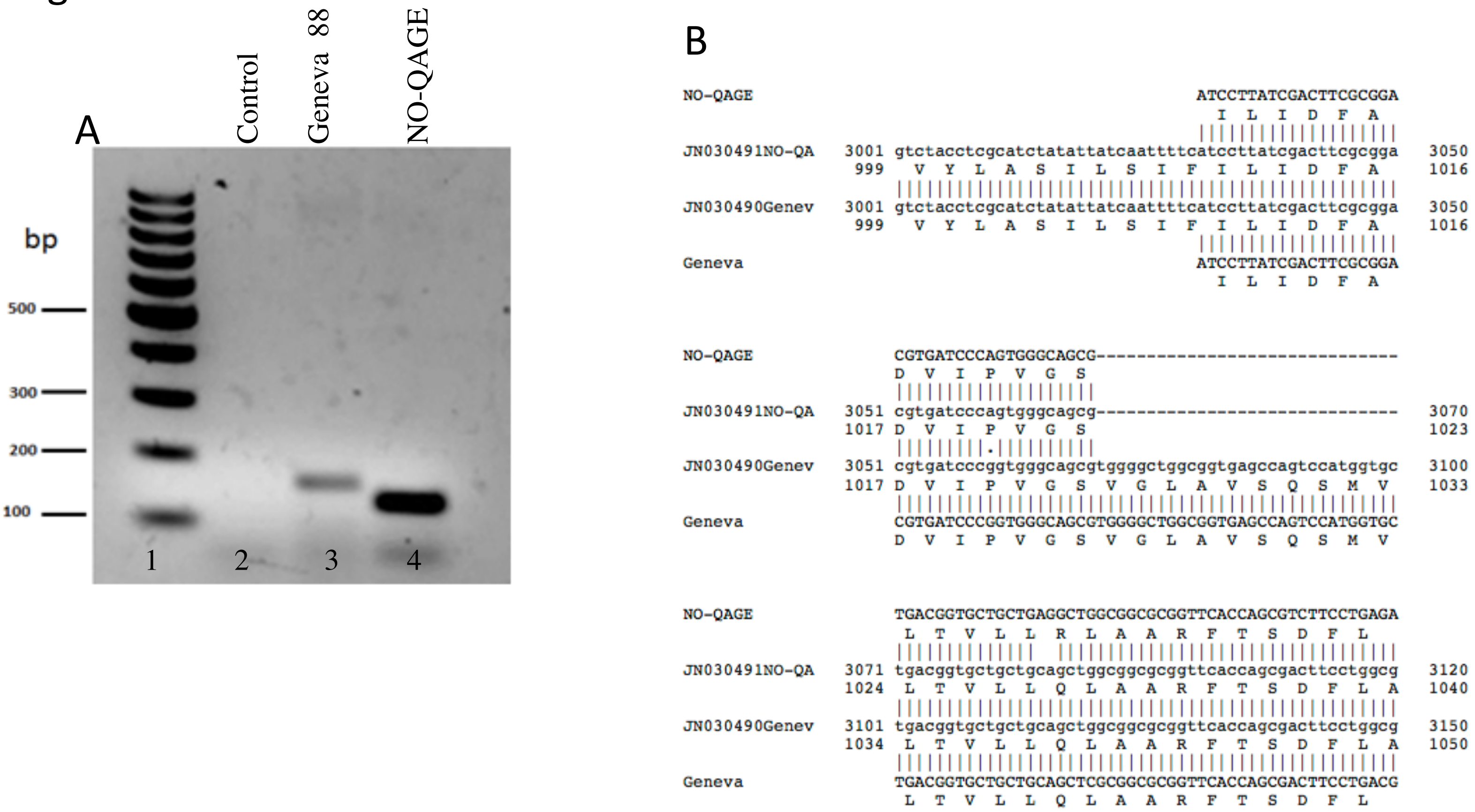

Fig S1. Characterization of ABCC2 mutant allele in NO-QAGE. cDNA was synthesized from RNA samples from 20 larvae from each strain and a 157 bp PCR product of exon 20 was amplified using primers described in Experimental Procedures. Panel A, agarose gel electrophoresis of the PCR products obtained from cDNA of Geneva 88 (lane 3) or NO-QAGE (lane 4). Lane 1, MW marker; lane 2, PCR reaction without RNA sample. Panel B, DNA sequence alignment of the PCR products from Geneva 88 and NO-QAGE compared to the previously reported sequence of both alleles labeled by the Genbank accession numbers of NO-QAGE (JN030491) and Geneva 88 (JN030490) (25).

Table S1. Scanning densitometry data for comparing oligomer formation in resistant (NO-QAGE) and susceptible (Geneva 88) strains of *P. xylostella*

Fig. 2 Oligomerization of Cry1Ac associated with BBMV

Replicate	Optical density		
	Geneva 88	AZP-R	(NO-QAGE/Geneva 88) X 100%
1	37036	12579	34.0 %
2	38534	19478	50.5 %
3	32025	3261	10.2 %
Mean	35865	11772	31.6 %
Std dev			11.7%

Fig. 2 Oligomerization of Cry1AcMod associated with BBMV

Replicate	Optical density		
	Geneva 88	NO-QAGE	(NO-QAGE/Geneva 88) X 100%
1	51918	59166	114 %
2	54347	53295	98.1 %
3	52261	46264	88.5 %
Mean	63508	14204	100.2 %
Std dev			7.4 %

Fig. 3 Oligomerization of Cry1Ac in the presence of BBMV

Replicate	Optical density		
	Geneva 88	NO-QAGE	(NO-QAGE/Geneva 88) X 100%
1	30499	11120	36.5 %
2	37446	10008	26.7 %
3	35474	1543	4.4 %
Mean	34473	7557	22.5 %
Std dev			9.5 %

Fig. 4 Insertion of pre-formed oligomers into BBMV

Replicate	Optical density		
	Geneva 88	NO-QAGE	(NO-QAGE/Geneva 88) X 100%
1	21279	18800	88.4 %
2	17383	12460	71.7 %
3	16474	17359	105.4 %
Mean	18378	16206	88.5 %
Std dev			16.4 %

Fig. 4 Insertion of pre-formed oligomers (1/2) into BBMV

Replicate	Optical density		
	Geneva 88	NO-QAGE	(NO-QAGE/Geneva 88) X 100%
1	12327	6989	56.7 %
2	8113	4859	59.9 %

3	5605	2873	51.3 %
Mean	8681	4907	56.0 %
Std dev			17.4 %

Fig. 4 Insertion of pre-formed oligomers (1/5) into BBMV

Replicate	Optical density		
	Geneva 88	NO-QAGE	(NO-QAGE/Geneva 88) X 100%
1	10684	4761	44.6 %
2	8702	969	11.1 %
3	10455	3033	29.0 %
Mean	9947	2921	28.2 %
Std dev			18.6 %
